# Genetic Diversity and Breeding Signatures for Regional *Indica* Rice Improvement in Guangdong of Southern China

**DOI:** 10.1186/s12284-023-00642-3

**Published:** 2023-05-16

**Authors:** Yu Hang, Liu Yue, Sun Bingrui, Liu Qing, Mao Xingxue, Jiang Liqun, Lyu Shuwei, Zhang Jing, Chen Pingli, Pan Dajian, Chen Wenfeng, Fan Zhilan, Li Chen

**Affiliations:** 1 Rice Research Institute, Guangdong Academy of Agricultural Sciences, Guangzhou, 510640 China; 2 Key Laboratory of Genetics and Breeding of High Quality Rice in Southern China (Co-construction by Ministry and Province), Ministry of Agriculture and Rural Affairs, Guangzhou, 510640 China; 3 Guangdong Key Laboratory of New Technology in Rice Breeding, Guangzhou, 510640 China; 4 Guangdong Rice Engineering Laboratory, Guangzhou, 510640 China

**Keywords:** Rice, Yield improvement, Resequencing, Breeding signature, GWAS

## Abstract

As the pioneer of the Green Revolution in China, Guangdong province witnessed the improvement and spread of semi-dwarf *Xian*/*Indica* rice cultivars and possessed diverse rice germplasm of landrace and cultivars. A total of 517 accessions containing a core germplasm of 479 newly sequenced landraces and modern cultivars were used to reveal breeding signatures and key variations for regional genetic improvement of *indica* rice from Guangdong. Four subpopulations were identified in the collection, which including Ind IV as a novel subpopulation that not covered by previously released accessions. Modern cultivars of subpopulation Ind II were inferred to have less deleterious variations, especially in yield related genes. About 15 Mb genomic segments were identified as potential breeding signatures by cross-population likelihood method (XP-CLR) of modern cultivars and landraces. The selected regions spanning multiple yield related QTLs (quantitative trait locus) which identified by GWAS (genome-wide association studies) of the same population, and specific variations that fixed in modern cultivars of Ind II were characterized. This study highlights genetic differences between traditional landraces and modern cultivars, which revealed the potential molecular basis of regional genetic improvement for Guangdong *indica* rice from southern China.

**Supplementary Information:**

The online version contains supplementary material available at 10.1186/s12284-023-00642-3.

## Background

Rice (*Oryza sativa*) feeds more than half of the world’s population, and rice yield is vital for world food security. Rice genetic improvement in China has facilitated the increase of its production over the past several decades. Guangdong of southern China witnessed the breeding, introduction and spread of semi-dwarf *indica* rice accessions. Since then, rice yield has been increased by about three-fold with the breakthrough of high-yield rice cultivars. However, with the spread of modern cultivars, rice landraces that were grown by local farmers is gradually disappearing. For the goal of further production increasing, the usage of genetic diversity for the valuable germplasm needs to be enhanced in breeding programs. Tremendous efforts have been made by germplasm scientist for the collection and conservation of landraces and locally-improved traditional cultivars of southern China. These landraces and cultivars represent the rice genetic diversity of southern China before and after the rice “Green Revolution”, which could be used to reveal genetic trajectory for regional *indica* rice breeding and phenotype enhancement. Revelation of the functional variations related to the success of rice breeding in southern China will promote the utilization of genetic resources for future rice breeding. Moreover, characterization of the genome sequences, genetic diversity and functional variations of these germplasm collections is becoming very critical for the next potential breakthrough of rice production.

The advancement of sequencing technologies enabled the analysis of genetic diversity for large collection of germplasm, which promoted the revelation of domesticated loci, and accelerated the identification of functional genes. Genotypes of a large collection of 517 rice landraces were identified with onefold-coverage sequencing and accurate imputation method, population structure, genome-wide association analysis and haplotype analysis were conducted using about 3.6 million nonredundant SNPs (Huang et al. 2010). Thereafter, higher-depth sequencing with more than 15-fold coverage were conducted on 40 cultivated and 10 wild rice accessions to identify selection signatures during domestication using nucleotide polymorphisms (Xu et al. 2012), and larger collection of 446 wild diverse rice accessions and 1083 cultivated varieties were also genotyped by sequencing and used for the identification of 55 selective sweeps during domestication (Huang et al. 2012). Further, 10,074 F_2_ lines from 17 representative hybrid rice combinations were genotyped, and heterosis related loci were identified (Huang et al. 2016). Release of sequencing data from “3000 Rice Genomes Project” (3KRGP) largely facilitated the identification of untapped variations and novel genes (Fuentes et al. 2019; Wang et al. 2018). Jointly data analysis of 3KRGP and Indian long and short grain germplasm identified the long low-diversity region harboring key gene regulating grain weight (Kumar et al. 2020). Recently, the sequencing of local germplasm and improved varieties illustrated the regional genetic diversity in detail. Sequencing of 239 *japonica* rice elites from China, Japan and Korea identified 1131 novel genes and artificial selection signals (Liu et al. 2021). Analysis of 672 Vietnamese rice genomes described their classification and identified 21 unique QTLs from 19 traits (Higgins et al. 2021). Genotyping and systemically phenotyping of 200 *japonica* rice varieties grown in central China over the past 30 years revealed the genetic factors regulating the balance of yield, quality and blast resistance (Xiao et al. 2021).

Artificial selection and breeding signatures during the succession of rice varieties makes deep insight into their genetic improvement. Low-coverage sequencing of 1479 landrace and modern cultivars from 73 countries revealed 200 regions were differentially selected between two major *indica* subpopulations, and yield was correlated with number of the signatures (Xie et al. 2015). Locally selective sweeps also showed pressure during artificial selection and farmer cultivation. Genotyping by sequencing of 108 core on-farm conserved rice landraces from Yunnan revealed 186 and 183 potential selective-sweep between different collection date (Cui et al. 2019). Different selection signature during breeding indicated by genetic differentiation between early and late cultivars of *indica* and *japonica* in Taiwan (Hour et al. 2020). As the pioneer of “Green Revolution” for *indica* rice in China, Guangdong province have rich diversity of germplasms. However, large-scale population genomics of rice landraces and improved varieties for the study of genetic diversity and identification of regional breeding signatures of are still lacking.

In this study, core germplasm of locally planted rice accessions of landrace and cultivars from Guangdong of southern China before and after rice “Green Revolution” were collected. Agronomic traits were systematically investigated by field experiments, and they were genotyped by 10-fold depth genome resequencing. Genetic diversity was analyzed and breeding signatures for modern cultivar subpopulation were identified and annotated by QTLs of eleven agronomic traits. Specific genetic variations and favorable alleles that fixed in subpopulation of modern cultivar were identified that can be used for molecular marker assisted breeding.

## Results

### Genetic Diversity and Population Structure Analysis

A total of 517 accessions consisting mainly of *indica* rice germplasm, which including 358 landrace and 159 artificially improved cultivars from Guangdong, China were used to identify genomic variations (Additional file 1: Table S1). The 479 newly-sequenced accessions generated 24.23 million 100 bp pair-end sequencing reads for each accession. Quality assessment of these sequencing reads revealed the average Q30 base quality (99.9% base call accuracy) percent was 99.65% (Additional file 1: Table S2). The average mapping depth against the MSU7 reference genome was 12.02 with 91.68% coverage ratio (Additional file 1: Table S3). Averagely 2.04 million SNPs, 82.34 thousand insertions and 127.99 thousand deletions were identified for 517 *indica* rice accessions (Additional file 1: Table S4).

**Fig. 1:**
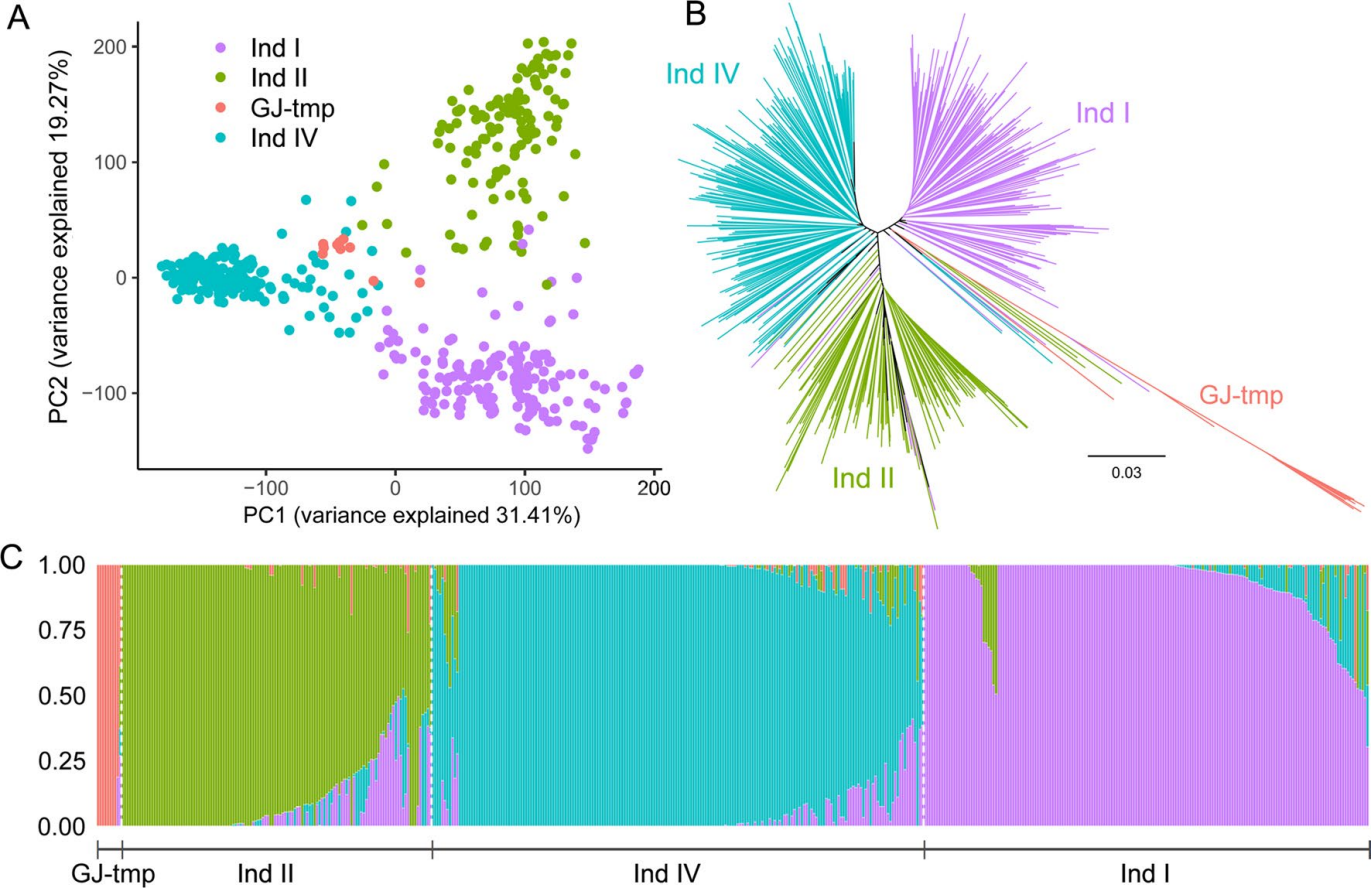
Population structure analysis of Guangdong *indica* rice accessions. PCA plot (**A**), phylogenetic analyses (**B**) and population structure (**C**) showing genetic diversity and clustering of all accessions based on whole genome SNP variations. For PCA plot, PC1 (principal component 1) and PC2 (principal component 2) are showed on horizontal and vertical axes, and percentages of variance explained were noted in parentheses

**Fig. 2:**
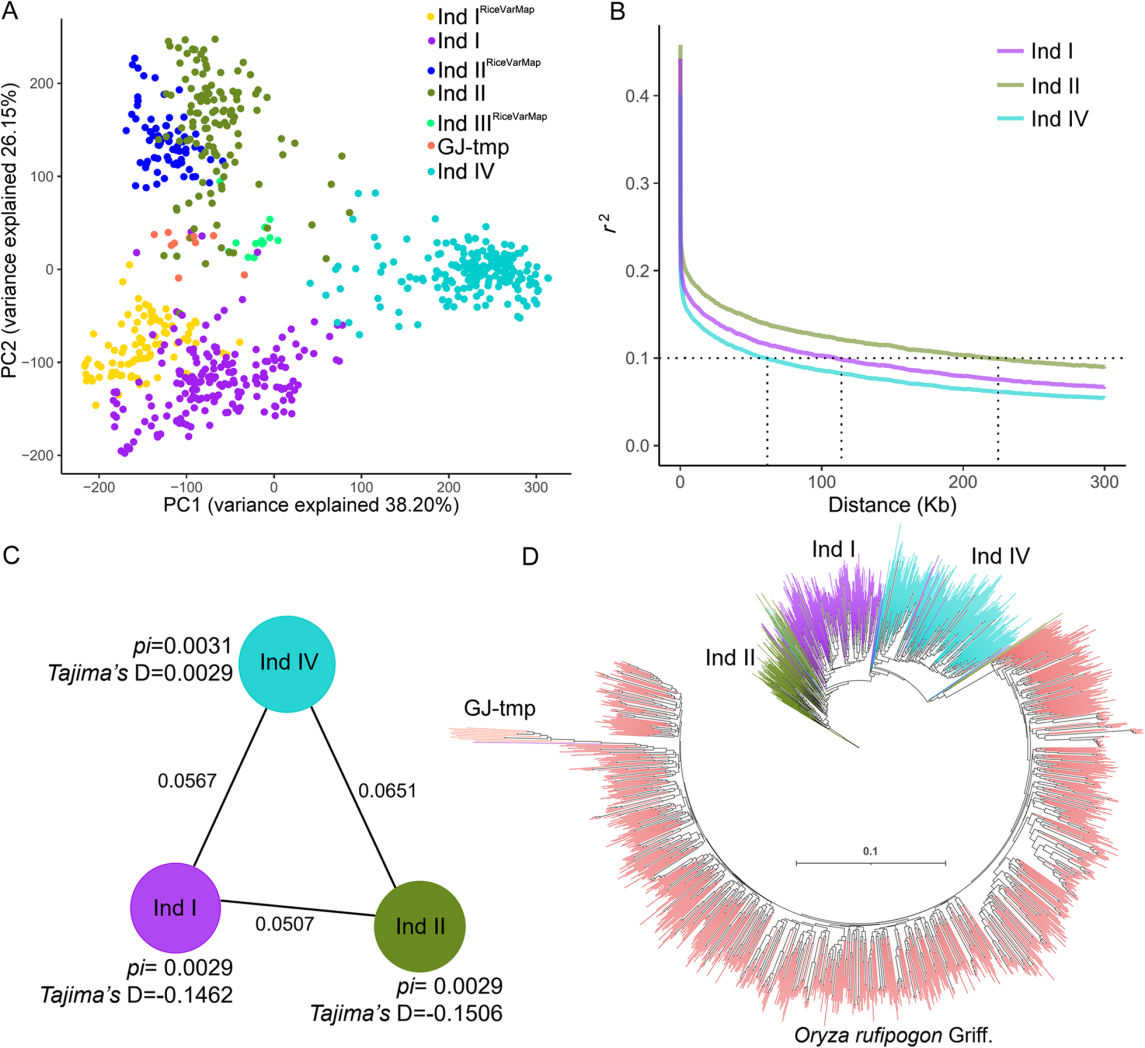
Genetic diversity and phylogenetic relationship of subpopulations for Guangdong *indica* rice. **A** PCA plot depicting the comparison of genetic clusters of 479 Guangdong and 220 *indica* accessions from RiceVarMap database. **B** Linkage disequilibrium (LD) decay analysis of three main subpopulations. **C** Genetic diversity and differentiation of three subpopulations for Guangdong *indica* rice. The size of the circles represents the level of genetic diversity (*pi*) of the subpopulations, and, and length of lines represent *fst* values between subpopulations. **D** Phylogenetic tree for Guangdong *indica* rice and common wild rice (*Oryza rufipogon* Griff.)

Population structure analysis was conducted by principal components analysis (PCA), phylogenetic and admixture analysis. In admixture analysis, group 1 contained 211 accessions (201 landraces and 10 cultivars), and group 2 contained 306 accessions (157 landraces and 149 cultivars) when subpopulation number (*k*) was 2. And three groups, namely group 1 (20 landraces and 116 cultivars), group 2 (189 landraces and 11 cultivars) and group 3 (149 landraces and 32 cultivars) can be identified when *k* = 3 (Additional file 2: Fig. S1). Integrated with PCA (Fig. 1A), phylogenetic tree (Fig. 1B) and admixture analysis (Fig. 1C), a total of 4 subpopulations were finally determined. With the reference of 182 accessions (with three subpopulation that named with Ind I, Ind II and Ind III) from RiceVarMap database, and the phylogenetic relationship with accessions from 3KRG, these 4 subpopulations were named as Ind I (landrace), Ind II (cultivar), Ind IV (landrace) and GJ-tmp in this study (Fig. 2A, Additional file 2: Fig. S2). GJ-tmp diverged from other subpopulations, and most of accessions from GJ-tmp subpopulation were glutinous rice landraces. Another subpopulation Ind I contained a total of 181 accessions, 151 accessions of which were landraces and 30 cultivars that were bred before 1980s. Subpopulation Ind II contained 126 accessions, which have 117 cultivars and 9 landraces. Subpopulation Ind IV have 189 landraces and 11 cultivars that were bred before 1980s (Additional file 1: Table S1). A recently released and refined *indica* reference genome (9311) was also used to call genetic variations and conduct population structure analysis, which obtained similar results for genetic clustering for all those accessions (Additional file 2: Fig. S3).

The length of linkage disequilibrium (LD) decay for subpopulation Ind I, Ind II and Ind IV other than the glutinous rice subpopulation GJ-tmp were estimated using the square of the correlation coefficient (*r*^2^) between variations. LD decay distance for Ind IV, Ind I and Ind II were 61.0 kb, 110.1 kb and 219.8 kb, respectively. The extension of LD decay distance for Ind II indicated that the cultivar subpopulation Ind II underwent artificial selection pressure during the process of genetic improvement. Interestingly, the landrace subpopulation Ind I probably have selection effect by regional farmer breeders as its LD decay distance longer than landrace subpopulation Ind IV (Fig. 2B). Genetic diversity (*pi* and *Tajima’s* D) and differentiation (*fst*) analysis were conducted for three main subpopulations of Guangdong *indica* rice. The *pi* values for Ind IV, Ind I and Ind II were 0.0031, 0.0029 and 0.0029, respectively. Ind IV have higher *pi* values, while Ind I and Ind II have similar values. The *Tajima*’s D value for Ind IV was positive, while they were negative for Ind I and Ind II, which implying potential selection effect in subpopulation of Ind I and Ind II. Genetic divergence (*fst*) between Ind IV and Ind I is smaller than that of Ind IV and Ind II, which indicates Ind II was higher diverged from Ind IV than Ind I (Fig. 2C). Phylogenetic tree with 998 common wild rice (*Oryza rufipogon* Griff.) lines also indicates degree of differentiation from wild rice populations from high to low was Ind IV, Ind I and Ind II (Fig. 2D). Together with these results, we deduced that the genetic differences of these regionally cultivated rice lines were attribute to the cultivation period, as modern cultivars may have high speed and flexible distance in their seed dispersal.

### Phenotypic Comparison for Subpopulations

The selection pressure by local breeders during rice improvement for the past half century largely changed the agronomic traits between traditional and modern rice accessions. Genetic diversity and LD decay analysis indicates potential selection pressure in Ind I, and even stronger selection effect in modern cultivar subpopulation Ind II. The alteration of agronomic traits for these subpopulations recorded the trajectory of these selection effect. A total of eleven important agronomic including plant height (PH), heading date (HD), yield per plant (YPP), panicle number (PN), grain number per panicle (GNPP), seed setting (SS), thousand grain weight (TGW), panicle length (PL), grain length (GL), grain width (GW) and grain length width ratio (GLWR) were investigated and analyzed.

**Fig. 3:**
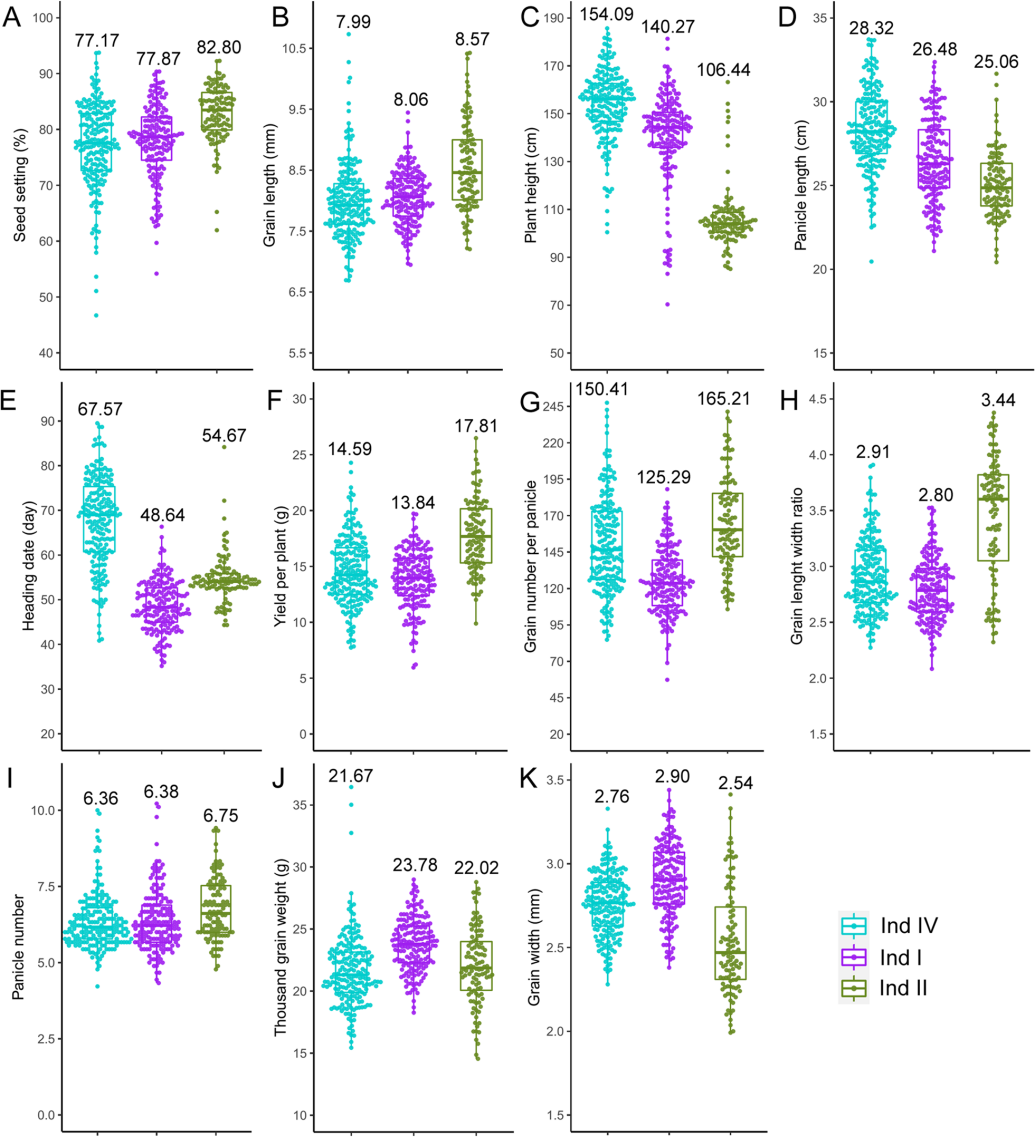
Phenotype comparison of eleven main agronomic traits for three main subpopulations of Guangdong *indica* rice. Subpopulations were ordered by LD decay distance from short to long, and average trait values for each subpopulation were noted above boxplots

During the improvement progress of Ind IV, Ind I and Ind II, values of eleven agronomic traits showed four different types of changing trends. Seed setting rate (Fig. 3a) and grain length (Fig. 3b) were increased during modern breeding process, as shown by the comparison of Ind IV, Ind I and Ind II. Plant height (Fig. 3c) and panicle length (Fig. 3d) descended during this process, which represents the main phenotype alteration for semi-dwarf rice cultivars that released during rice “green revolution” of southern China. Trait of heading date (Fig. 3e), yield per plant (Fig. 3f), grain number per panicle (Fig. 3g) and grain length width ratio (Fig. 3h) showed fluctuation of decline in Ind I and elevation in Ind II. Panicle number (Fig. 3i), thousand grain weight (Fig. 3j) and grain width (Fig. 3k) were raised in Ind I but decreased in Ind II. The increasing of thousand grain weight and grain length and grain width reflecting the selection of high yield rice lines with large grain size, while the breeding and application of high-quality “Simiao rice” with small and slender grains, the Guangdong *indica* rice showed decrease of these traits and the increase of grain length width ratio.

### Frequency of Deleterious or Beneficial Allele During Genetic Improvement

**Fig. 4:**
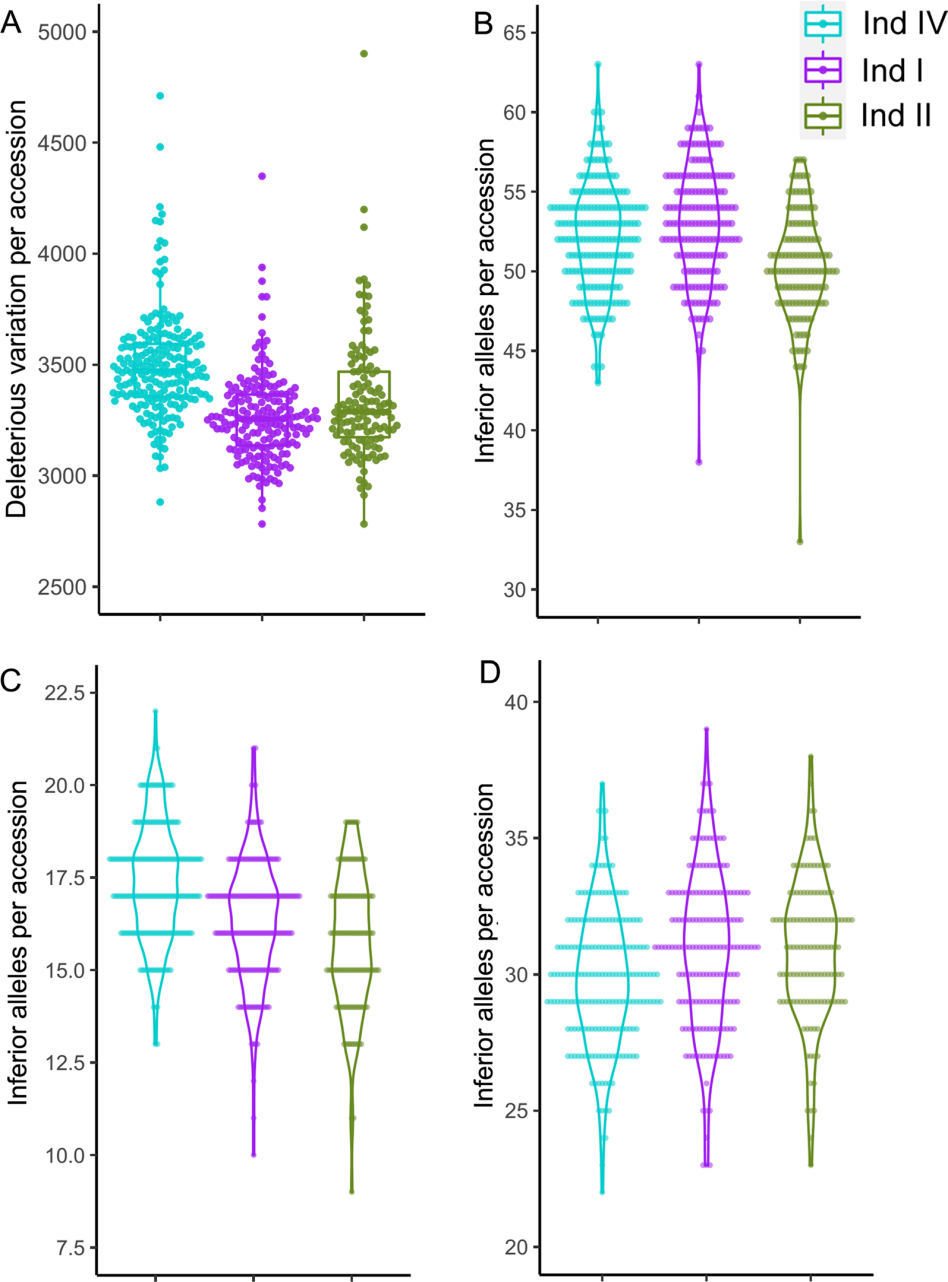
Deleterious variations in accessions of three main subpopulations. **A** Number of deleterious variations that predicted by SIFT (sorting intolerant from tolerant) software. Number of inferior alleles of all genes (**B**), yield related genes (**C**) and stress related genes (**D**) that annotated using RiceNavi database. Subpopulations were ordered by LD decay distance from short to long

Number of deleterious variations that encode adverse amino acid were predicted in three main subpopulations, and the number of accessions from landrace subpopulation Ind I and Ind IV were compared cultivar subpopulation Ind II. Firstly, deleterious variations identified by SIFT software showed the total count of deleterious variations were stepwise decreasing in Ind I (median number was 3255.0) and Ind II (median number was 3287.5) compared with Ind IV (median number was 3472.0), which implying these variations were lost during modern cultivars improvement under artificial selection pressure (Fig. 4A and Additional file 1: Table S5). Secondly, a total of 319 quantitative trait nucleotides (QTNs) of the 212 vital gene in rice of RiceNavi database were used to annotate accessions of three subpopulations (Additional file 1: Table S6). For all genes, the average inferior allele count of accessions in Ind IV, Ind I and Ind II were 52.19, 52.96 and 50.32, respectively. Inferior allele counts were 17.38, 16.34 and 15.72 for yield related genes and 29.83, 30.91 and 30.88 for Ind IV, Ind I and Ind II, respectively. These results suggesting that modern cultivars of subpopulation Ind II accumulated favorable alleles, especially for yield related genes during improvement (Fig. 4B and C). However, modern cultivars lost some favorable allele of stress responsive genes (Fig. 4D).

### QTLs and Breeding Signatures of Guangdong Indica Rice

Breeding signatures of modern cultivar subpopulation Ind II were identified using significant distorted patterns in allele frequency of XP-CLR method. Modern cultivar subpopulation Ind II was respectively compared with landrace subpopulation Ind IV and Ind I. For the comparison of Ind IV and Ind II, a total of 150 genomic segments spanning 15.10 Mb potentially selected genomic regions by modern cultivar breeding were identified (Additional file 1: Table S7). And for the comparison of Ind I and Ind II, a total of 146 genomic segments with genomic length of 14.59 Mb were identified (Additional file 1: Table S8).

Genome wide association study (GWAS) were conducted for eleven yield and yield-related traits and the effects of candidate genes were identified. For instance, *Ghd7.1*/*DTH7* explained 9.50% of heading date variances, *sd1* explained 15.07% of plant height variances, *GS3* explained 4.57%, 9.84% and 6.46% phenotype variances for thousand grain weight, grain length and grain length width ratio, *GSE5* explained 11.40%, 22.85% and 11.49% phenotype variances for thousand grain weight, grain width and grain length width ratio, and GS5 explained 40.70% and 4.37% phenotype variances for grain width and grain length width ratio (Additional file 1: Table S9, Additional file 2: Fig. S4). Effect of allele combination were analyzed for plant height and grain size genes. Average plant height of accessions with combination of *sd1*^Hap1^ and *Oshox4*^Hap2^ was 116.49 cm, which significantly lower than 151.70 cm of *sd1*^Hap1^ and *Oshox4*^Hap1^ (Additional file 1: Table S10). A total of 25 major allele combinations of grain size genes *GS3*, *GSE5* and *GS5* were detected. Thousand grain weight ranged from 19.56 to 24.22 g, grain length ranged from 7.62 to 9.43 mm, grain width ranged from 2.30 to 2.96 mm, and grain length width ratio ranged from 2.66 to 3.99 for the accessions with the 25 allele combinations. For instance, the high quality “Simiao” rice Meixiangzhan2hao have the combination of *GS3*^Hap3^, *GSE5*^Hap2^ and *GS5*^Hap6^ with thousand grain weight of 20.88 g and grain length width ratio of 3.99 (Additional file 1: Table S11). The phenotype effects of known genes that genotyped by RiceNavi were also evaluated, and several QTNs have potential effects on Guangdong indica rice phenotype. For instance, two variations of *Hd1* gene (9338004 and 9338220 on Chr6) shows effect to promoting heading date by about 9 days (Additional file 1: Table S12). Four genes that regulating eating quality were also genotyped. Ten accessions were genotyped to have fragrance allele of *Badh2* gene, and 9 of which are Ind II accessions. The only different site of two elite cultivars Huanghuazhan and Meixiangzhan2hao was the presence and absence of the fragrance allele of *Badh2* gene (Additional file 1: Table S13).

**Fig. 5:**
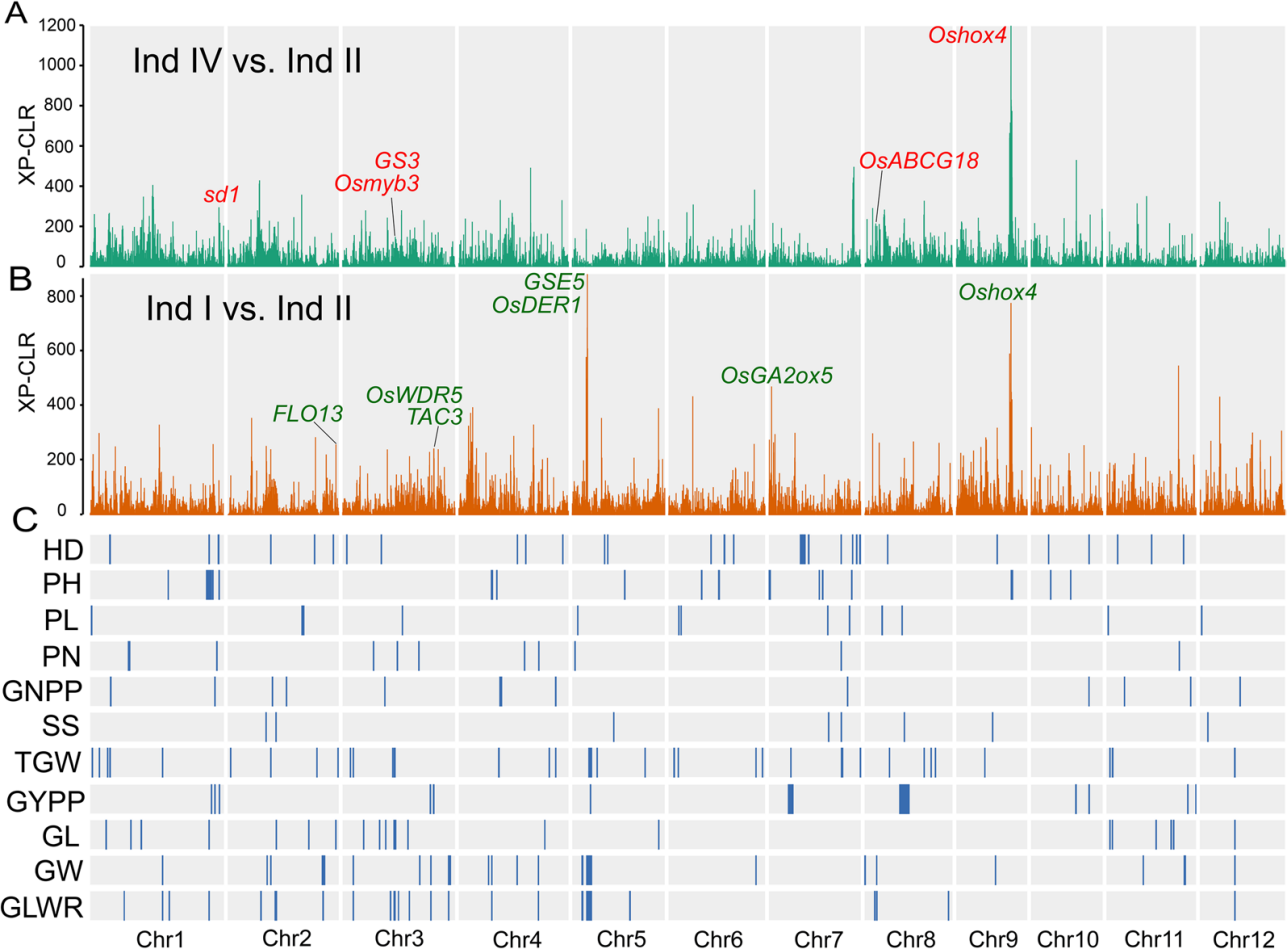
Selection sweeps for modern cultivar Ind II and the annotation of breeding signatures by QTLs of eleven agronomic traits. Distribution of XP-CLR scores between modern cultivar Ind II and local landrace subpopulations of Ind IV (**A**) and Ind I (**B**). **C** Annotation of breeding signatures using QTLs identified by GWAS

XP-CLR analysis were conducted by comparing Ind II with Ind IV (Fig. 5A) and Ind I (Fig. 5B), and the QTLs were further used to annotate the regions of breeding signatures (Fig. 5C). A total of 24 and 23 intersections between agronomic QTLs and selected genomic regions for subpopulation Ind IV and Ind I, respectively. Known vital yield and yield-related genes under selection pressure were detected. For instance, *sd1*, *Oshox4* and *OsGA2ox5* were found under selection of plant height, *OsWDR5*, and *TAC3* were selected for the improvement of grain yield per plant, and favorable alleles of *GS3*, *Osmyb3* and *FLO13* were selected for grain length. Interestingly, different selected genes were detected for subpopulation Ind IV and Ind II. Plant height genes *sd1* and *Oshox4* were selected in Ind IV, and the selected genes in Ind I were *OsGA2ox5* and *Oshox4*. For grain yield per plant, *TAC3* and *OsWDR5* were under selection pressure in Ind I but not in Ind IV. *GS3* was selected for grain length in Ind IV but not in Ind I. *GSE5* and *OsDER1* were selected for grain width and grain length width ratio in subpopulation Ind I, but *OsABCG18* was selected in Ind IV (Fig. 5).

### Allele Fixation During the Breeding Process of Guangdong Indica Rice

**Fig. 6:**
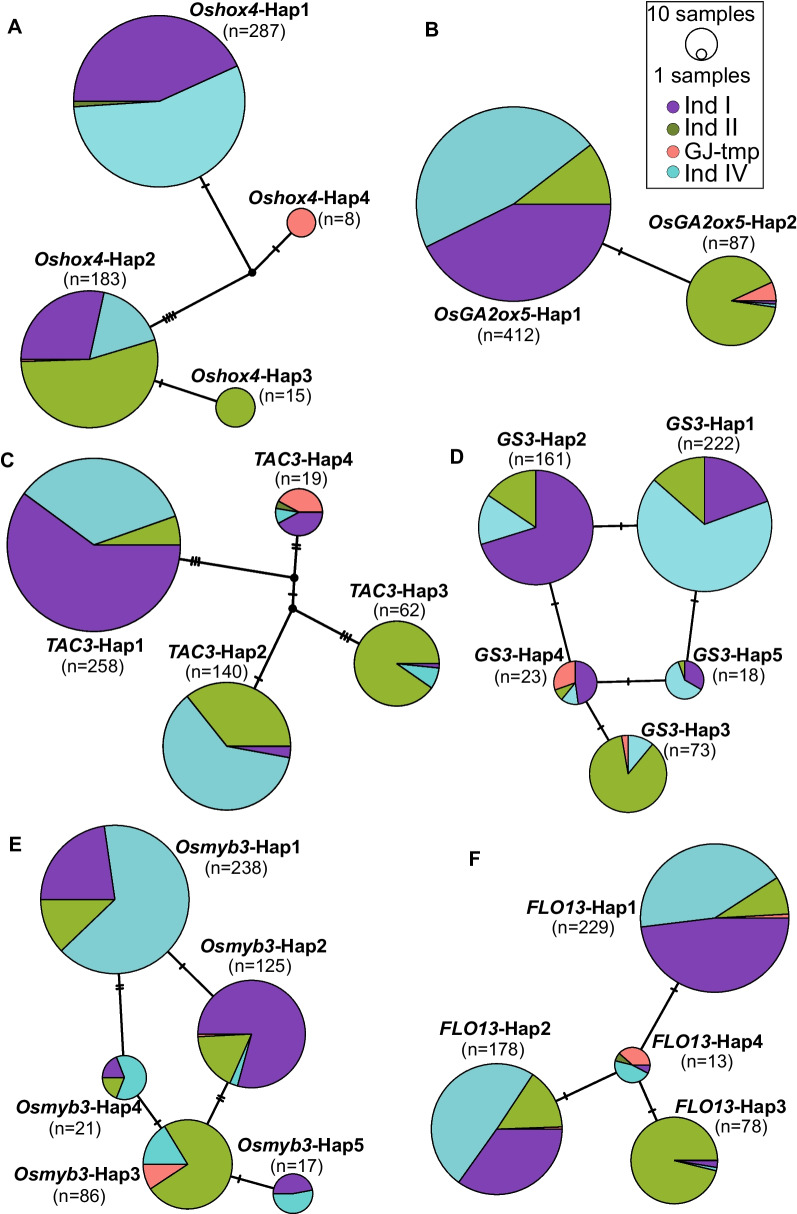
Haplotype networks of six key yield related genes in selected regions. **A** *Oshox4* related to plant height, **B** *OsGA2ox5* related to plant height, **C** *TAC3* related to tiller angle, **D** *GS3* related to grain length and weight, **E** *Osmyb3* related to grain length, and **F** *FLO13* related to grain weight. Circle size is proportional to accession number of one haplotype. Number of ticks in network edges represents the variation counts between haplotypes

**Fig. 7:**
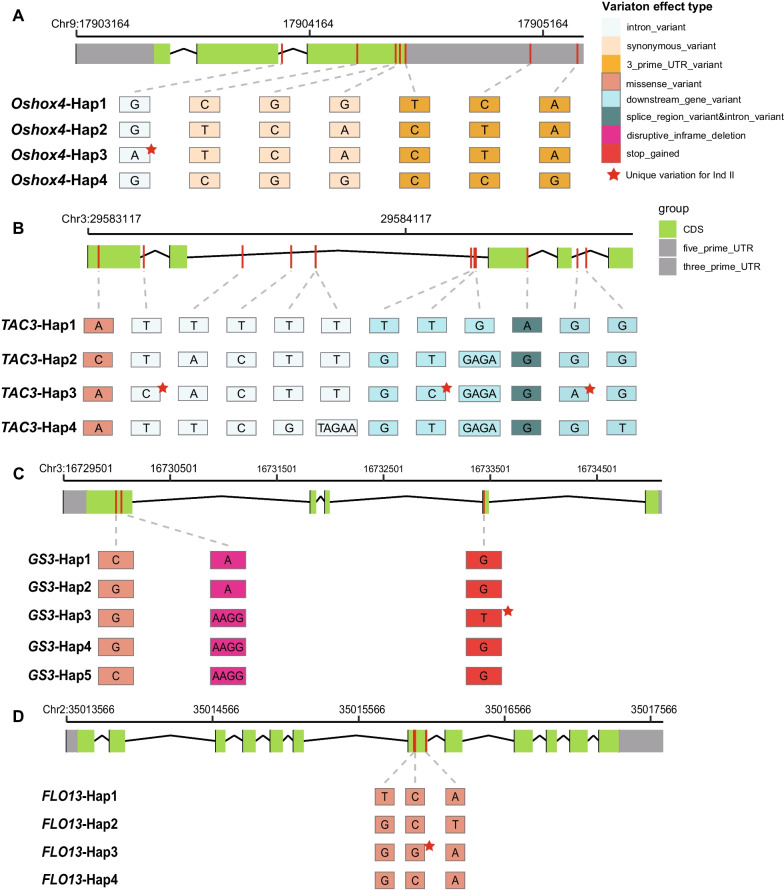
Gene structure and haplotype specific variations of *Oshox4* (**A**), *TAC3* (**B**), *GS3* (**C**) and *FLO13* (**D**) for subpopulation Ind II. Background colors of bases represent annotation and effect level of the variation. Red stars mark the specific variations for the haplotype of Ind II

Gene haplotype analysis were conducted to illustrate evolutionary relationship and identify selected alleles during modern breeding and improvement of subpopulation Ind II. A total of 6 potentially favorable alleles were fixed in Ind II for key yield related genes, those genes were *Oshox4* and *OsGA2ox5* for plant height, *TAC3* for tiller angle and yield, *GS3*, *Osmyb3* and *FLO3* for grain size and weight. Four main haplotypes were identified for plant height gene *Oshox4*, haplotype network analysis revealed that *Oshox4*-Hap3 (Ind II fixed haplotype) was derived from *Oshox4*-Hap2, following the variations of *Oshox4*-Hap1 (Ind IV and Ind I) and *Oshox4*-Hap4 (GJ-tmp). For two main haplotypes of plant height gene *OsGA2ox5*, most accessions of Ind II have *OsGA2ox5*-Hap2, while landraces of subpopulation Ind I and Ind IV have *OsGA2ox5*-Hap1. Tiller angle and yield related gene *TAC3* have four main haplotypes in these accessions, and *TAC3*-Hap3 are a fixed haplotype of Ind II modern cultivars. Grain length and weight gene *GS3* have five main haplotypes, *GS3*-Hap3 was fixed during breeding and improvement process for Ind II. Hap3 of grain length gene *Osmyb3* was mainly selected for Ind II during modern rice breeding by one and two variations from *Osmyb3*-Hap4 and *Osmyb3*-Hap2. For the four haplotypes of grain weight gene *FLO13*, Hap3 is a predominant allele in Ind II, which was selected from *FLO13*-Hap4 with one missense variation (Fig. 6). In those modern cultivar fixed alleles, six Ind II specific variations were identified. *Oshox4*-Hap3 have one specific intron variation between exon 1 and exon 2, *TAC3*-Hap3 have three cultivar specific variations, *GS3*-Hap3 have one stop codon gained variation, and *FLO13*-Hap3 have one specific missense variation in Ind II (Fig. 7).

## Discussion

The major breakthrough of “green revolution” leads quantum leaps of rice productivity (Cheng et al. 2020), intensive breeding efforts and artificial selection have facilitated the significant improvement of *indica* rice yield in Guangdong, where the “green revolution” started in China. Unlike previously reported selection analysis of rice accession from multiple geographic positions (Li et al. 2020a, b; Lv et al. 2020; Xie et al. 2015; Xu et al. 2016; Ye et al. 2022), we focusing on the locally adaptative selection of rice in Guangdong by comparing the genomic variations and agronomic traits of locally cultivated landraces by farmers before “green revolution” and modern improved cultivars. For example, modern breeding of rice quality started from the end of last century in Guangdong favors slender grain type for regional appetite in southern Aisa, which makes the increase of grain length and width ratio, and decrease of grain weight in subpopulation of modern cultivars.

The artificial replacement of deleterious variations with favorable alleles during breeding and improvement are meaningful to meet social demands for crop and food. By integrating GWAS for agronomic traits and breeding signatures for selected regions, regionally selected key genes that influencing yield and yield related traits for Guangdong *indica* rice were identified. *Oshox4* was identified as a selected gene for plant height in our accessions, which plays negative function in gibberellin responses and influencing plant height and tiller number (Dai et al. 2008; Zhou et al. 2015). An Ind II specific haplotype (*Oshox4*-Hap3) were identified for cultivars. Another regulator for rice growth and architecture, *OsGA2ox5*, were also identified as selected gene of plant height for cultivar subpopulation Ind II when compared with Ind I (Lo et al. 2008), and *OsGA2ox5*-Hap2 was a selected haplotype by cultivars from Ind II. Tiller angle gene *TAC3* were selected for yield per plant of Ind II when compared with Ind I, and *TAC3*-Hap3 were selected by cultivars from Ind II (Dong et al. 2016). *GS3* and *Osmyb3* were identified to be selected for Ind II when compared with Ind IV, and the Hap3 of these two genes were selected for cultivars (Li et al. 2020a, b; Fan et al. 2006; Mao et al. 2010). Starch biosynthesis and grain weight gene *FLO13* was selected for Ind II compared with Ind I, and *FLO13*-Hap3 with a unique missense variant in cultivars was a potential selected haplotype (Hu et al. 2018). These selected favorable haplotypes are promising functional alleles regulating the yield improvement of Guangdong modern cultivars.

## Conclusions

In summary, large-scale genomic and yield assessment of Guangdong landrace and modern cultivars promotes analysis of their diversity, classification and phylogenetic relationship. We revealed less deleterious variations number in modern cultivars than landrace. Selected genomic regions were also identified and annotated using GWAS of vital agronomic traits, which leads the identification of selected key genes during Guangdong rice breeding and improvement. These results shed light on regionally breeding trajectory and artificial selection, and provides valuable resources for rational design of molecular breeding.

## Materials and Methods

### Field Experimental Design and Phenotyping of Agronomic Traits

Agronomic traits of 479 accessions of Guangdong rice core germplasm was investigated on experimental field of Rice Research Institute, Guangdong Academy of Agricultural Sciences for two seasons of 2019 and 2021 under conventional field management. All accessions were planted in 1437 blocks under randomized complete block design with three replications. A total of eleven agronomic traits were investigated and calculated. Plant height (PH) was measured as length from the ground to the highest point of the plant, heading date (HD) was recorded when half of the plants in a block have reached the heading stage and days from sowing to heading is calculated, yield per plant (YPP) was the total weight of filled grains per plant, panicle number (PN) was the number of effective panicles per mature plant, grain number per panicle (GNPP) was the mean total number of grains per panicle on a single plant, seed setting (SS) was calculated as the percentage of filled grains to total grains per plant, grain length (GL), grain width (GW) and panicle length (PL) were measured when seeds are mature (Yu et al. 2020). Thousand grain weight (TGW) were calculated by the division of total weight to total filled grain number, and grain length width ratio (GLWR) was the division of GL to GW.

### Genomic Resequencing and Variation Calling

Young leaves of 358 landrace and 121 improved cultivars of *indica* rice accessions from Guangdong province of southern China were collected to construct sequencing libraries according to the manufacturer’s instructions, and qualified libraries were sequenced using Illumina HiSeq platform. A total of 38 Guangdong improved cultivars were collected from the NCBI SRA database with accession numbers of PRJNA321462, PRJNA522896 and PRJNA656900 (Additional file 1: Table S1). Quality of raw sequencing data were accessed using FastQC (v0.11.9) software (Andrews 2010), and low-quality data were trimmed using TrimGalore (version 0.6.6) to generate clean sequencing data. Clean data were mapped onto reference genome (MSU7) using BWA (0.7.17-r1188) software with default parameter (Li and Durbin 2009). MarkDuplicates in Picard (2.12.1) was used to eliminate PCR duplication and sorting BAM files, and genomeCoverageBed of bedtools (v2.27.1) was used to calculate genome coverage ratios. SNPs (single nucleotide polymorphisms) and InDels (insertions and deletions) were then called using HaplotypeCaller of Genome Analysis Toolkit (GATK, version 4.2.2.0) pipeline (McKenna et al. 2010), and annotated using SnpEff (4.3 s) with the GFF3 file of MSU7 reference genome (Cingolani et al. 2012).

### Population Structure Analysis

Principal components analysis (PCA), phylogenetic and admixture analysis were employed to classify subpopulations (Yu et al. 2021). For population structure analysis, SNP variations were filtered using VCFtools (0.1.16) software with parameter “–max-missing 0.95” and “–maf 0.05”. PCA method with kmeans clustering algorithm in CropGBM software was used to reducing dimensions of genotypic data (Yan et al. 2021), and eigenvalues were calculated by plink software. Phylogenetic relationship was constructed using VCF2Dis software (https://github.com/BGI-shenzhen/VCF2Dis) and illustrated by using FigTree (v1.4.3) software (https://github.com/rambaut/figtree). ADMIXTURE (version 1.3.0) software (Alexander and Lange 2011) was used to analyze population structure with *k* values ranged from 2 to 12. The ancestry distributions of individuals were visualized using R script. Genetic diversity (*pi* and *Tajima’s* D) and differentiation (*fst*) analysis were conducted using VCFtools (0.1.16) software with 100 kb sliding windows. Linkage disequilibrium (LD) decay for each subpopulation was estimated and plotted using PopLDdecay (Zhang et al. 2019).

National *indica* rice sequencing data from RiceVarMap database (Zhao et al. 2015), variations of 3024 3KRG accession from SNP-seek database (Locedie et al. 2017) and variations of 998 wild rice lines from our recently research (Zhang et al. 2022) that used to conduct population structure and phylogenetic analysis in this study were subjected to the same data processing pipeline. A recently released and refined *indica* 9311 reference genome was also used to check the results of population structure analysis (Wang et al. 2022).

### Estimation of Variation Effects and Deleterious Mutation Prediction

Functional alteration of genomic variations was predicted using Sorting Intolerant From Tolerant 4G (SIFT 4G) software (Vaser et al. 2016). Variations with SIFT scores smaller than 0.05 was considered as putatively deleterious variations. Allele function for known genes with vital role in rice were annotated using RiceNavi database (Wei et al. 2021). Advantage and inferior allele were determined by manually check of allele functional alteration and its corresponding trait. Number of inferior alleles for accessions from each subpopulation were counted and plotted using boxplot or violin plot in R.

### Identification of Breeding Signatures Using XP-CLR

Breeding signatures for artificial selection were identified by using the cross-population composite likelihood ratio test (XP-CLR) method (Chen et al. 2010) and its updated version of python module (https://github.com/hardingnj/xpclr). XP-CLR was conducted between subpopulations of landrace and cultivar with 10 kb sliding windows. Genomic segments with XP-CLR values above the 80th percentile was considered as putatively selected regions. Adjacent segments within 20 kb distance were then merged into longer blocks, and blocks shorter than 40 kb were filtered out as such short blocks unlikely selected during the short history of modern rice improvement breeding. Long blocks with top 1% values of XP-CLR scores were finally considered as selected regions.

### Genome-Wide Association Analysis

For genome-wide association analysis (GWAS), multi-sample VCF file of genomic variations was converted into plink file and variations were screened with parameters of “–geno 0.1 –mind 0.4 –maf 0.05”. PCA analysis were conducted by plink software with five major components (Purcell et al. 2007). Kinship analysis and GWAS were conducted using GEMMA software using filtered genotypes and eleven agronomic traits (Zhou and Stephens 2012).

### Gene Haplotype Reconstruction and Network Analysis

Software beagle (version 5.2) was used to impute missing genetic variations that generated by GATK (Browning et al. 2021). Genomic variations of selected genes were extracted based on the positions by using BCFTools (Li 2011). Haplotype network of these genes were constructed by our previously described method (Yu et al. 2021). Haplotype network was constructed and illustrated by Popart software (Leigh and Bryant 2015).

## Supplementary Information

**Additional file 1**: **Table S1**. Information for accessions used in this study. **Table S2**. Quality assessment of genome sequencing data. **Table S3**. Genome mapping quality and genome coverage of genome sequencing data. **Table S4**. Genomic variations for 517 indica rice accessions against the MSU reference genome. **Table S5**. Number of deleterious variations in subpopulation of landrace and cultivar. **Table S6**. Genotyping results 319 quantitative trait nucleotidesof the 212 vital gene in rice from RiceNavi database. **Table S7**. Genomic segments that identified as breeding signatures between Ind IV and Ind II. **Table S8**. Genomic segments that identified as breeding signatures between Ind I and Ind II. **Table S9**. QTLs and known genes that identified by GWAS of eleven agronomic traits for Guangdong indica rice. **Table S10**. Allele combinations of plant heightgenes of Guangdong indica rice germplasm. **Table S11**. Allele combinations of thousand grain weight, grain length, grain widthand grain length width ratiogenes of Guangdong indica rice germplasm. **Table S12**. Phenotypic effect assessment of known QTNs that genotyped by RiceNavi. **Table S13**. Genotyping of 4 eating quality genes.

**Additional file 2**: **Fig. S1**. Admixture analysis when subpopulation numberwas set to twoand three. Numbers of cultivarand landracewere noted in parentheses for each subgroup. **Fig. S2**. Population structure analysis of Guangdong indica rice with accessions from RiceVarMap2 and 3KRG database. **Fig. S3**. Population structure analysis of Guangdong indica rice accessions using indica rice 9311 as reference genome. **Fig. S4**. Manhattan plots for genome-wide association analysis of eleven agronomic traits.

## Data Availability

The raw reads of whole-genome resequencing were available at the NCBI Sequence Read Archive with accession ID PRJNA934413. The sequences and annotations of reference genome MSU7 is available from the websites http://rice.plantbiology.msu.edu/.

## Abbreviations

SNP: Single-nucleotide polymorphisms
PCA: Principal component analysis
XP-CLR: The cross-population composite likelihood ratio test
QTLs: Quantitative trait locus
GWAS: Genome-wide association studies
LD: Linkage disequilibrium
QTNs: Quantitative trait nucleotides
KRG: 3000 Rice genome project
