# Supplementary material for: Genetic Diversity and Breeding Signatures for Regional Indica Rice Improvement in Guangdong of Southern China
Source: Rice (N Y). 2023 May 16;16:25. doi: 10.1186/s12284-023-00642-3 (PMC10188715; doi:10.1186/s12284-023-00642-3)
Supplement: Supplementary file 2 — Additional file 2: Fig. S1. Admixture analysis when subpopulation numberwas set to twoand three. Numbers of cultivarand landracewere noted in parentheses for each subgroup. Fig. S2. Population structure analysis of Guangdong indica rice with accessions from RiceVarMap2 and 3KRG database. Fig. S3. Population structure analysis of Guangdong indica rice accessions using indica rice 9311 as reference genome. Fig. S4. Manhattan plots for genome-wide association analysis of eleven agronomic traits. [file 12284_2023_642_MOESM2_ESM.docx]

**Genetic diversity and breeding signatures for regional *indica* rice improvement in Guangdong of southern China**

Yu Hang, Liu Yue, Sun Bingrui, Liu Qing, Mao Xingxue, Jiang Liqun, Lyu Shuwei, Zhang Jing, Chen Pingli, Pan Dajian, Chen Wenfeng, Fan Zhilan, Li Chen*

**Additional file 2:**


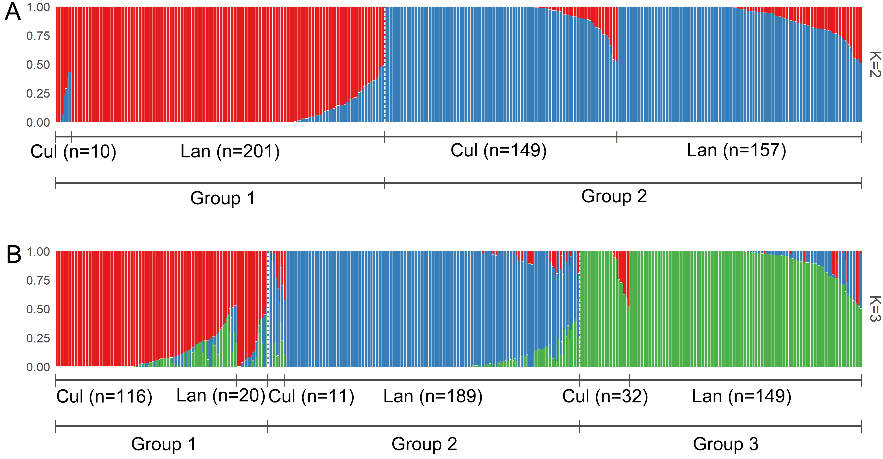


**Figure S1.** Admixture analysis when subpopulation number (*k*) was set to two (a) and three (b). Numbers of cultivar (Cul) and landrace (Lan) were noted in parentheses for each subgroup.


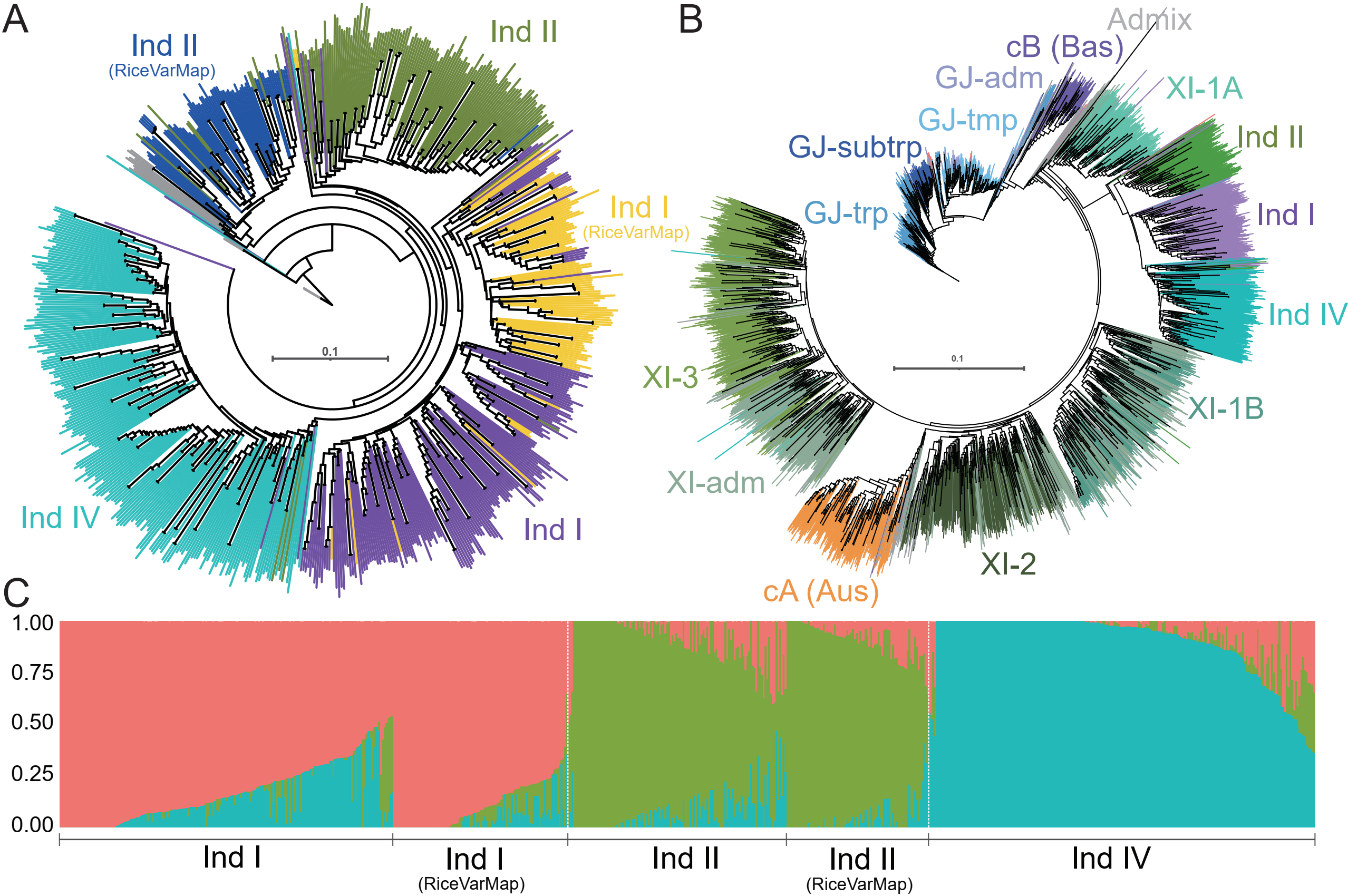


**Figure S2.** Population structure analysis of Guangdong *indica* rice with accessions from RiceVarMap2 and 3KRG database.

(A) Phylogenetic tree of Guangdong *indica* rice with *indica* rice accessions from RiceVarMap2, and the subgroups were named accordingly. (B) Phylogenetic tree of Guangdong *indica* rice with accessions from 3KRG, the subpopulation of glutinous or *japonica* rice accessions (orange colored) were grouped and named GJ-tmp refer to 3KRG. (C) Admixture plot of Guangdong *indica* rice with *indica* rice accessions from RiceVarMap2.


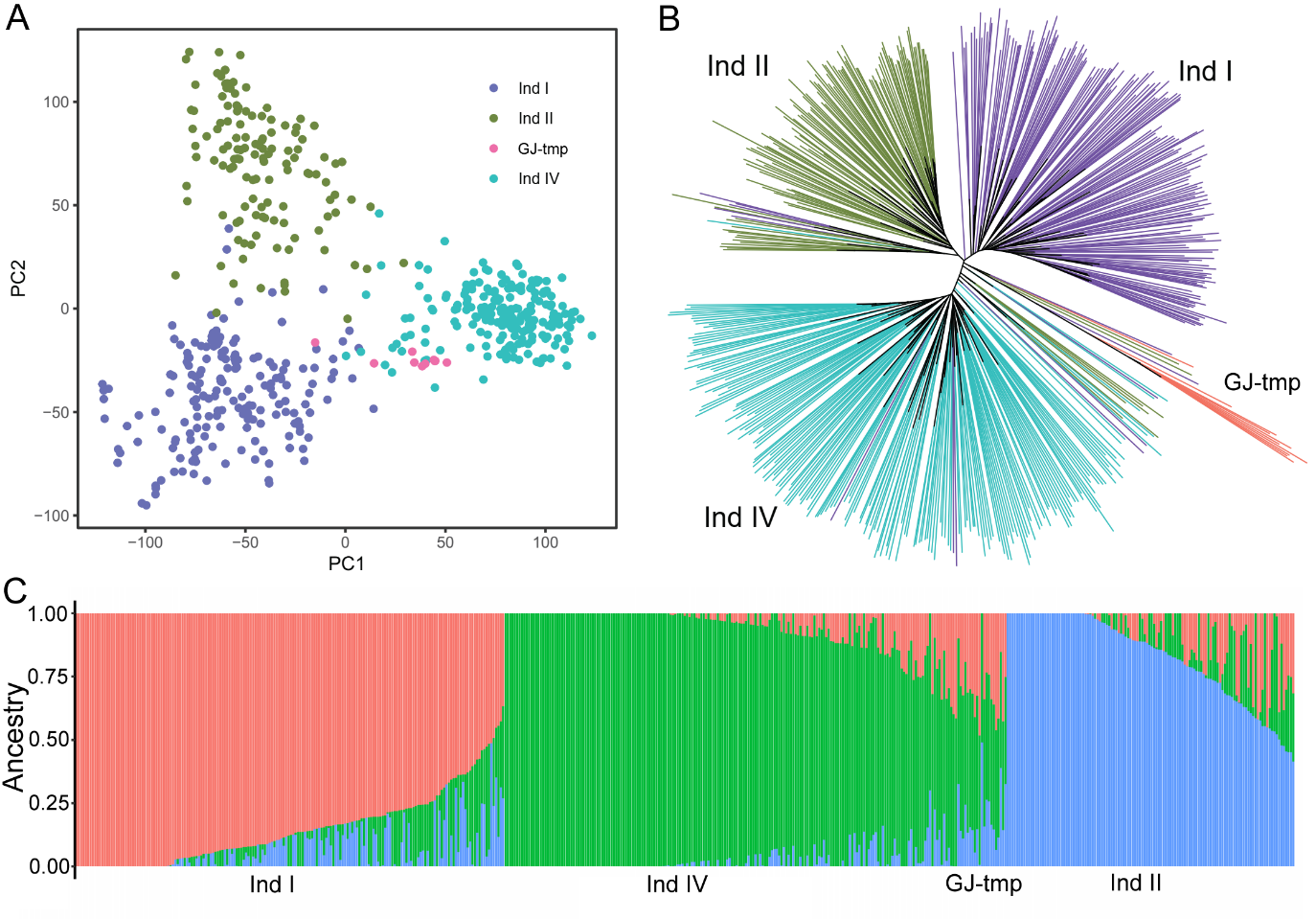


**Figure S3.** Population structure analysis of Guangdong *indica* rice accessions using *indica* rice 9311 as reference genome. PCA plot (a), phylogenetic analyses (b) and population structure (c) showing genetic diversity and clustering of all accessions based on whole genome SNP variations.


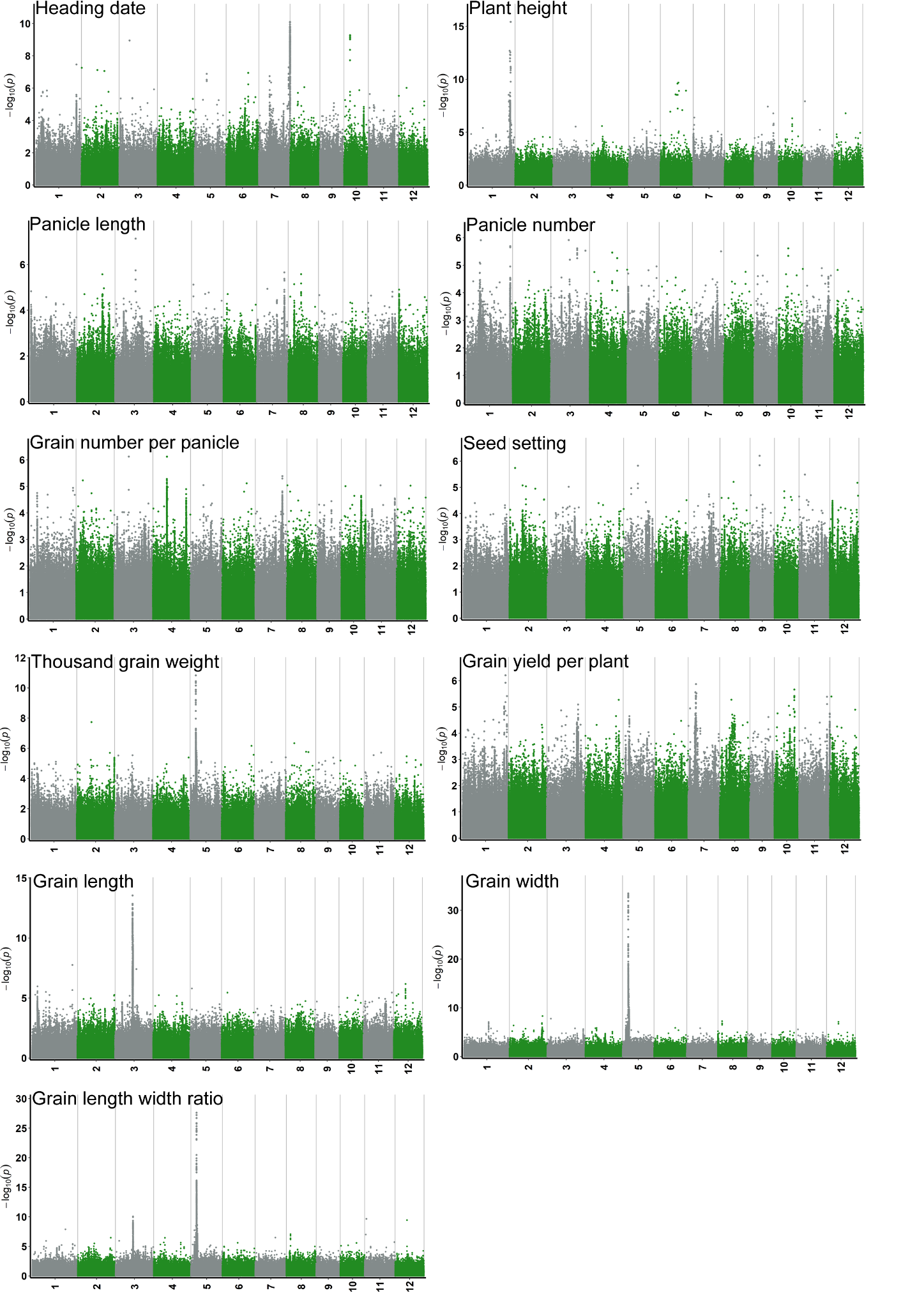
**Figure S4.** Manhattan plots for genome-wide association analysis of eleven agronomic traits.
